# Protective Effects of Several Common Amino Acids, Vitamins, Organic Acids, Flavonoids and Phenolic Acids against Hepatocyte Damage Caused by Alcohol

**DOI:** 10.3390/foods11193014

**Published:** 2022-09-28

**Authors:** Yashen Wang, Nanhai Zhang, Jingxuan Zhou, Peng Sun, Liang Zhao, Feng Zhou

**Affiliations:** 1 Beijing Key Laboratory of Functional Food from Plant Resources, College of Food Science and Nutritional Engineering, China Agricultural University, Beijing 100083, China; 2 Beijing Advanced Innovation Center for Food Nutrition and Human Health, Beijing Engineering and Technology Research Center of Food Additives, School of Food and Health, Beijing Technology and Business University, Beijing 100048, China

**Keywords:** ethanol, acetaldehyde, functional component, cell membrane damage

## Abstract

With the increase in alcohol consumption, more and more people are suffering from alcoholic liver disease (ALD). Therefore, it is necessary to elaborate the pathogenesis of ALD from the aspects of alcohol metabolism and harm. In this study, we established an alcoholic liver injury model in vitro by inducing L02 cells with different concentration of ethanol and acetaldehyde. Results showed that the metabolism of ethanol can promote the content of ROS, MDA, TNF-α, IL-6, and caspase 3, causing oxidative and inflammatory stress and membrane permeability changes. However, unmetabolized ethanol and acetaldehyde had little effect on cell membrane permeability and inflammation, indicating that ethanol metabolites were the main reason for cell membrane damage. We also evaluated the effects of amino acids (taurine and methionine), vitamins (E and vitamin D), organic acids (malic acid and citric acid), flavonoids (rutin and quercetin), and phenolic acids (ferulic acid and chlorogenic acid) on alcohol-induced cell membrane damage of L02 cells. Chlorogenic acid, taurine, vitamin E, and citric acid had remarkable effects on improving cell membrane damage. Malic acid, rutin, quercetin, and ferulic acid had obvious therapeutic effects, while vitamin D and methionine had poor therapeutic effects. The relationship between the structure and effect of active ingredients can be further studied to reveal the mechanism of action, and monomers can be combined to explore whether there is a synergistic effect between functional components, in order to provide a certain theoretical basis for the actual study of liver protection.

## 1. Introduction

Ethanol is widely present in alcoholic beverages, such as liquor, wine, and premixed liquor [1]. Alcohol intake is the main cause of alcoholic liver disease [2]. Globally, alcohol use accounts for approximately 3 million deaths every year and the overall burden of disease and injuries remains high [3]. Compared with poor areas, the prevalence in economically developed areas is higher [4]. Ethanol can be completely absorbed within 20–60 min after entering the digestive tract [5]. Drinking while fasting is conducive to alcohol absorption [6]. After drinking while fasting, the concentration of ethanol and acetaldehyde in blood can reach the maximum at about 1 h, and the concentration of ethanol is about 1000 times that of acetaldehyde and acetic acid [7]. After about 7 h of metabolism, the metabolism of ethanol and acetaldehyde in blood is complete [8]. Studies have found that the concentration of ethanol and acetaldehyde in blood determined by pharmacokinetics were 5.5 mM and 4.5 µM, respectively, after the human body ingested ethanol at 0.25 g/kg [9]. In addition, some studies have found that with the increase in alcohol consumption, blood alcohol concentration would gradually increase and showed a quasilinear correlation [10]. Ethanol is firstly metabolized to acetaldehyde in hepatocytes [11]. Ethanol is oxidized to acetaldehyde only through the alcohol dehydrogenase (ADH) pathway when the concentration of ethanol in blood is low [12]. However, as the concentration of ethanol in blood is increased, the microsomal ethanol oxidation system (MEOS) pathway and catalase pathway are also activated [13]. In normal humans, the ADH pathway accounts for more than 90%, while the MEOS pathway accounts for less than 10% [14]. However, when chronic alcohol disease occurred, MEOS is strengthened accordingly [15].

Free radicals generate during the metabolism of ethanol and acetaldehyde, which eventually leads to the damage of cell membrane structure [16] and also promotes the release of calcium ions, further activating caspase apoptotic protein cascade and causing cell apoptosis [17]. Free radicals can also cause nucleoside acid–base modification and DNA strand breakage [18]. In addition, oxidative stress induced by ethanol can cause the production of inflammatory factors [19]. After excessive alcohol consumption, ethanol leads to the production of a large amount of TNF-α, leading to inflammatory cascade, apoptosis, and damage [20,21]. Liver diseases caused by alcohol can be divided into alcoholic fatty liver, alcoholic hepatitis, alcoholic liver fibrosis, and alcoholic liver cirrhosis, and finally develop into alcoholic liver cancer in the later stage [22]. Excessive drinking can also cause damage to other organs, such as pancreas, cardiovascular, eyes, and brain [23].

Phenolic hydroxyl groups in polyphenols have strong affinity to hydrogen atoms or neutrons, which show antioxidant and protective effects against oxidative damage of biological tissue membrane and DNA caused by free radicals [24]. Organic acids can reduce the damage of oxidized low-density lipoprotein to cell membrane [25]. Vitamin E is a common lipophilic antioxidant and has a good scavenging effect on free radicals in the body [26]. Methionine has an important protective effect on liver injury [27]. Taurine has the function of scavenging oxygen free radicals, inhibiting lipid peroxidation, and stabilizing cell membrane [28]. Polyphenols, organic acids, vitamins, amino acids, and other bioactive components have shown certain protective effects on cell injury; however, there are few comparative studies of their effects.

In this study, ethanol, acetaldehyde, ethanol, and acetaldehyde combined with their metabolism inhibitors were induced to L02 cell line, which was designed to analyze the specific components that caused damage to hepatic cells and therefore established an alcoholic liver injury model in vitro. After that, amino acids (taurine and methionine), vitamins (E and vitamin D), organic acids (malic acid and citric acid) and polyphenols (rutin, quercetin, ferulic acid, and chlorogenic acid) were used to evaluate the protective effects on alcohol-induced cell membrane damage and apoptosis of L02 cells.

## 2. Materials and Methods

### 2.1. Chemicals

Taurine, methionine, vitamin E, vitamin D, malic acid, citric acid, rutin, quercetin, ferulic acid, and chlorogenic acid (98% pure) were obtained from Shanghai Yuanye Bio-Technology Co., Ltd. (Shanghai, China), and 3-[4,5-dimethylthiazol-2-yl]-2,5-diphenyltetrazoliumbromide (MTT), HEPES, 2′, 7′-dichlorofluorescin diacetate (DCFH- DA), and dimethyl sulfoxide (DMSO) were purchased from Sigma-Aldrich, Inc. (St. Louis, USA). Dulbecco’s modified Eagle’s medium (DMEM) with glucose, phenol red, fetal bovine serum (FBS), phosphate-buffered saline (PBS), penicillin, and streptomycin were purchased from Gibco Life Technologies (Grand Island, NY, USA). A bicinchoninic acid assay kit, trypsin, and NP-40 buffer were obtained from Beyotime Biotechnology Inc. (Beijing, China). All other chemicals used were of analytical grade and commercially available in China.

### 2.2. Cell Culture and Treatment

Human hepatic L02 cells were normal hepatocytes obtained from the China Cell Culture Center (Shanghai, China). L02 cells were cultured in DMEM supplemented with 10% FBS and 1% penicillin–streptomycin solution at 37 °C in a humidified atmosphere containing 5% CO_2_.

In the first experiment, L02 cells at 80% confluenc were exposed to ethanol, acetaldehyde, ethanol+4-methylpyrazole (4-MP, metabolic inhibitor of ethanol and acetaldehyde), and ethanol+acetaldehyde+4-MP for 1 h, 7 h, and 12 h.

In the second experiment, L02 cells at 80% confluency were exposed to 100 mM ethanol and ethanol combined with individual active compounds (taurine, methionine, vitamin E, vitamin D, malic acid, citric acid, rutin, quercetin, ferulic acid, and chlorogenic acid) for 12 h. The concentration and structure of active compounds are shown in Table 1.

### 2.3. Cell Viability Assay

The cell survival rate was determined using MTT assay. Briefly, after incubation with treatment medium, cells were washed with PBS and then incubated with 0.5 mg/mL MTT for 4 h at 37 °C in the dark. The supernatant was then removed, and 150 µL of DMSO was added to each well to dissolve the formazan crystals. A microplate reader (SpectraMaxM2e, Molecular Devices, CA, USA) was used to measure the absorbance value at 570 nm.

### 2.4. Status of Intracellular Oxidative Stress

Reactive oxygen species (ROS) production in L02 cells was detected by DCFH-DA fluorescent probes. In the present study, cells were plated in black 96-well plates and treated as described above. Then, the cells were incubated with DCFH-DA (25 µM) in HEPES balanced salt solution (pH 7.4) for 30 min at 37 °C in the dark. Eventually, cells were washed thoroughly with 200 µL HEPES balanced salt solution, and levels of intracellular ROS were determined by measuring the fluorescence intensity at 535 nm emission and 490 nm excitation using a SpectraMax M2e multifunctional microplate reader.

### 2.5. Activity of AST, ALT, and LDH Levels

After treatment, the medium was collected to measure the activities of aspartate transaminase (AST), alanine transferase (ALT) and lactate dehydrogenase (LDH) using corresponding commercial kits following the manufacturer’s instructions (Nanjing Jiancheng Bioengineering Institute, Nanjing, China).

### 2.6. Intracellular Levels of MDA, TNF-α, and IL-6

After treatment, L02 cells were washed with PBS and lysed with NP-40. The contents of malonaldehyde (MDA), interleukin-6 (IL-6), and tumor necrosis factor α (TNF-α) were analyzed using corresponding ELISA kits (Keyingmei Biotechnology and Science Inc., Beijing, China) according to the manufacturer’s instructions. The results were normalized to the total cellular protein, which was measured via bicinchoninic acid assay using the commercial kit (Beyotime Biotechnology Inc., Beijing, China).

### 2.7. Caspase 3 Activity Assay

Caspase 3 activity was analyzed by using a caspase 3 activity assay kit (Beyotime Biotechnology Inc., Beijing, China) according to the manufacturer’s instructions.

### 2.8. Membrane Potential

L02 cells were seeded in six-well plates and treated by different compounds. After incubation for 12 h, the medium was removed and the cells washed twice with PBS. Subsequently, cells were incubated with DIBAC4(3) (5 µM) for 30 min at 37 °C. Fluorescence was measured using a SpectraMax M2e multifunctional microplate reader at 530 nm with excitation at 488 nm.

### 2.9. Statistical Analysis

Data differences were evaluated with *t*-test using GraphPad Prism 8.0 (GraphPad, San Diego, CA, USA). Data graphics were constructed using GraphPad Prism 8.0 (GraphPad, San Diego, CA, USA). Results are expressed as means ± SD with significance accepted at *p* < 0.05.

## 3. Results

### 3.1. Effect of Metabolic Inhibitors on Cell Relative Survival Rate

**Figure 1:**
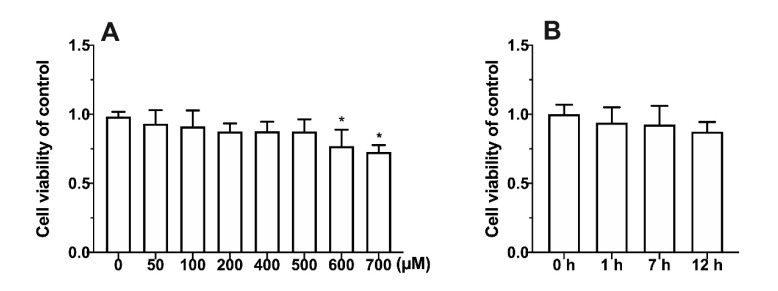
Effect of different concentrations (**A**) and induction time (**B**) of metabolic inhibitors on cell relative survival rate. Values represent means ± SD (n ≥ 6). * *p* < 0.05 compared with the control group.

From Figure 1A, compared with the 0 µM group, 500 µM 4-MP did not cause significant damage to cells. However, when the concentration was over 500 µM, the relative survival rate of cells was significantly affected (*p* < 0.05). In addition, with the treatment of 500 µM 4-MP for 12 h, no significant change of cell viability occurred (Figure 1B). Therefore, 500 µM 4-MP was used for further experiments.

### 3.2. Effects of Four Different Treatments on Cell Proliferation

**Figure 2:**
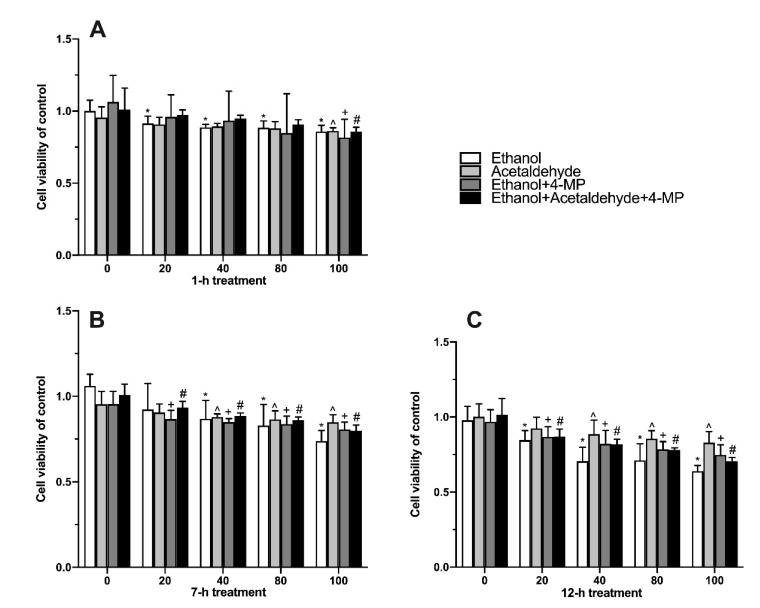
Effect of four different treatments on cell proliferation in L02 cell lines treated for 1 h (**A**), 7 h (**B**), and 12 h (**C**). Values represent the means ± SD (n ≥ 6). * *p* < 0.05 compared with 0 mM ethanol, ^ *p* < 0.05 compared with 0 µM acetaldehyde, + *p* < 0.05 compared with 0 mM ethanol + 500 µM 4-MP, # *p* < 0.05 compared with 0 mM ethanol + 0 µM acetaldehyde + 500 µM 4-MP.

The relative survival rate of cells is an index reflecting the toxicity of samples to cells. As shown in Figure 2A, compared with 0 µM group, treatment with 20 mM ethanol for 1 h significantly affected the cell viability of L02 cells (Figure 2A). When the time of treatment was extended to 7 h, 40 mM ethanol, ethanol+4-MP. and ethanol+acetaldehyde+4-MP significantly damaged cells in comparison with the 0 µM group (Figure 2B). With the increase in induction time (12 h), the effect of ethanol on cell viability was gradually more severe (Figure 2C).

### 3.3. Effects of Four Different Treatments on Intracellular MDA Production

**Figure 3:**
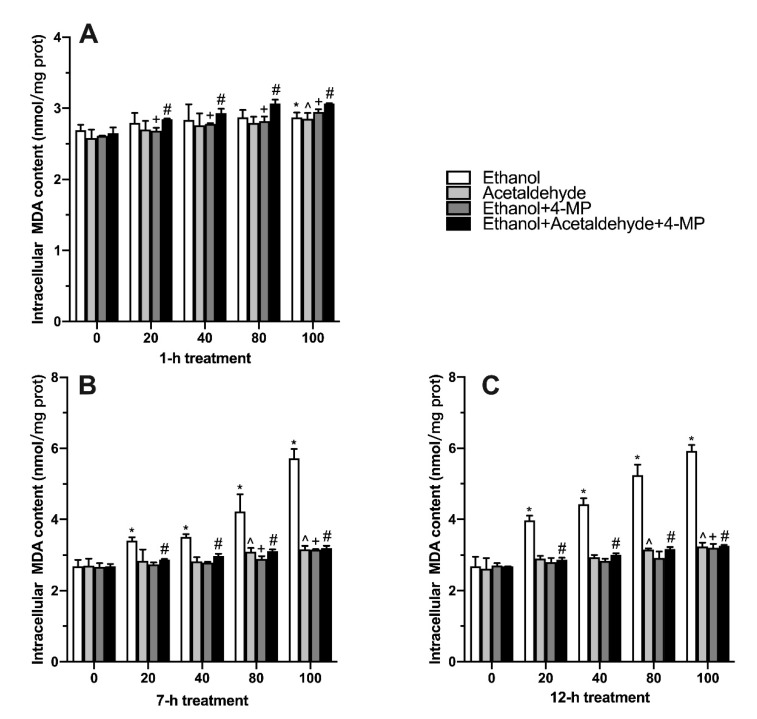
Effect of four different treatments on malondialdehyde content in L02 cell lines treated for 1 h (**A**), 7 h (**B**), and 12 h (**C**). Values represent means ± SD (n ≥ 6). * *p* < 0.05 compared with 0 mM ethanol, ^ *p* < 0.05 compared with 0 µM acetaldehyde, + *p* < 0.05 compared with 0 mM ethanol + 500 µM 4-MP, # *p* < 0.05 compared with 0 mM ethanol + 0 µM acetaldehyde + 500 µM 4-MP.

MDA is an index reflecting the level of lipid peroxidation. It is shown in Figure 3A that with the concentration of ethanol, acetaldehyde, ethanol+4-MP or ethanol+ acetaldehyde+4-MP increased, the intracellular MDA level elevated. When the time of treatment was extended to 7 and 12 h, 20 mM of ethanol significantly elevated the content of MDA (Figure 3B,C).

### 3.4. Effects of Four Different Treatments on Intracellular Oxidative Stress

**Figure 4:**
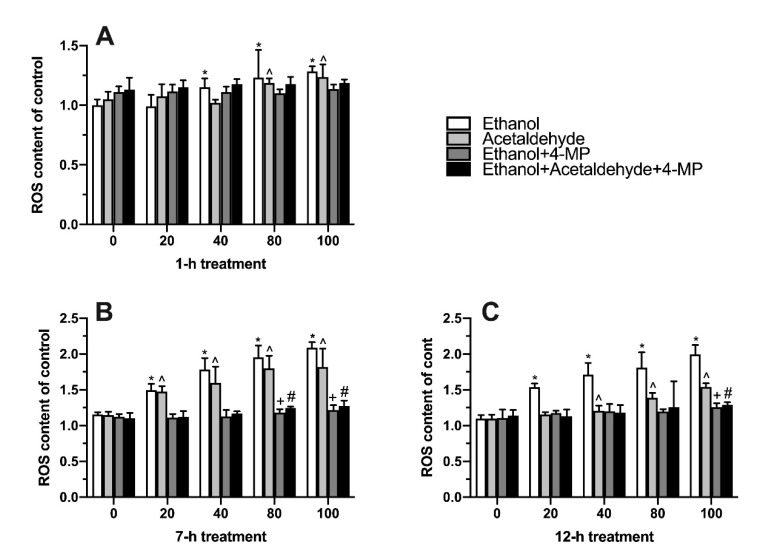
Effect of four different treatments on reactive oxygen species content in L02 cell lines treated for 1 h (**A**), 7 h (**B**), and 12 h (**C**). Values represent means ± SD (n ≥ 6). * *p* < 0.05 compared with 0 mM ethanol, ^ *p* < 0.05 compared with 0 µM acetaldehyde, + *p* < 0.05 compared with 0 mM ethanol + 500 µM 4-MP, # *p* < 0.05 compared with 0 mM ethanol + 0 µM acetaldehyde + 500 µM 4-MP.

ROS are oxidative free radicals produced by the body that can reflect the state of oxidative stress. It is shown in Figure 4A that the ROS level in 40, 80, and 100 mM ethanol groups significantly increased after 1 h of induction compared with the 0 mM ethanol group. In the acetaldehyde group, intracellular ROS content also significantly increased at 80 mM and 100 mM. The acetaldehyde+4-MP group showed no significant changes in ROS levels at 1 h treatment. When treatment time was increased to 7 h, ethanol significantly caused the accumulation of ROS from 20 mM to 100 mM, and the same trend occurred in the acetaldehyde group, which also induced oxidative stress in L02 cells (Figure 4B). When L02 cells were continuously induced for 12 h, the content of oxygen free radicals in the ethanol group was still higher than the other groups (Figure 4C).

### 3.5. Effects of Four Different Treatments on TNF-α Production

**Figure 5:**
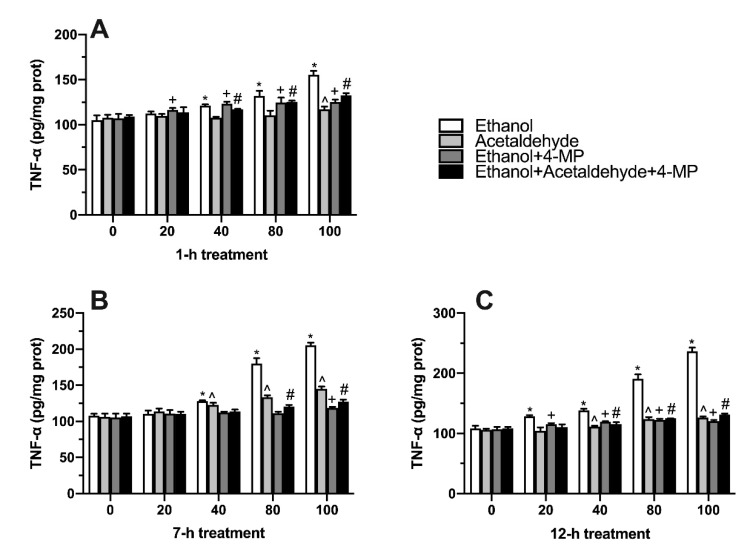
Effect of four different treatments on TNF-α content in L02 cell lines treated for 1 h (**A**), 7 h (**B**), and 12 h (**C**). Values represent means ± SD (n ≥ 6). * *p* < 0.05 compared with 0 mM ethanol, ^ *p* < 0.05 compared with 0 µM acetaldehyde, + *p* < 0.05 compared with 0 mM ethanol + 500 µM 4-MP, # *p* < 0.05 compared with 0 mM ethanol + 0 µM acetaldehyde + 500 µM 4-MP.

The content of TNF-α was measured to evaluate the inflammatory response of L02 cells treated by ethanol, acetaldehyde, or their metabolic inhibitor. As shown in Figure 5A, compared with 0 mM ethanol group, TNF-α level in 40, 80, and 100 mM ethanol group significantly increased at 1 h treatment. After treatment for 7 h, the level of TNF-α also significantly increased in the ethanol group from a concentration of 40 mM to 100 mM. Acetaldehyde significantly increased the content of TNF-α at the concentration of 40 µM, 80 µM, and 100 µM compared with the control group (Figure 5B). With the treatment time increased to 12 h, the effect of ethanol and acetaldehyde on intracellular TNF-α was like that of 7 h induction (Figure 5C).

### 3.6. Effects of Four Different Treatments on IL-6 Production

**Figure 6:**
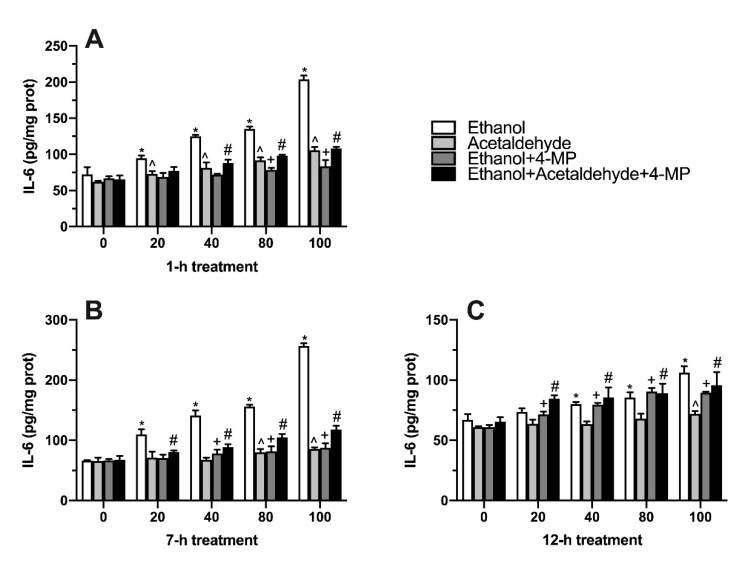
Effect of four different treatments on IL-6 content in L02 cell lines treated for 1 h (**A**), 7 h (**B**), and 12 h (**C**). Values represent means ± SD (n ≥ 6). * *p* < 0.05 compared with 0 mM ethanol, ^ *p* < 0.05 compared with 0 µM acetaldehyde, + *p* < 0.05 compared with 0 mM ethanol + 500 µM 4-MP, # *p* < 0.05 compared with 0 mM ethanol + 0 µM acetaldehyde + 500 µM 4-MP.

IL-6 is also an index reflecting the level of inflammation. Figure 6A showed that compared with the 0 mM ethanol group, the concentration of IL-6 in the ethanol group increased significantly in all ethanol and acetaldehyde groups. When the treatment lasted 7 h, the ethanol group also showed a significant difference from the dose of 40 mM to 100 mM compared with the control group (Figure 6B). In addition, 100 mM ethanol caused a severe inflammatory response in L02 cells. After 12 h treatment, the effect of ethanol and acetaldehyde on intracellular IL-6 was like that of 7 h induction (Figure 6C).

### 3.7. Effects of Bioactive Components on Ethanol-Induced Cell Proliferation, Transaminase and LDH

**Figure 7:**
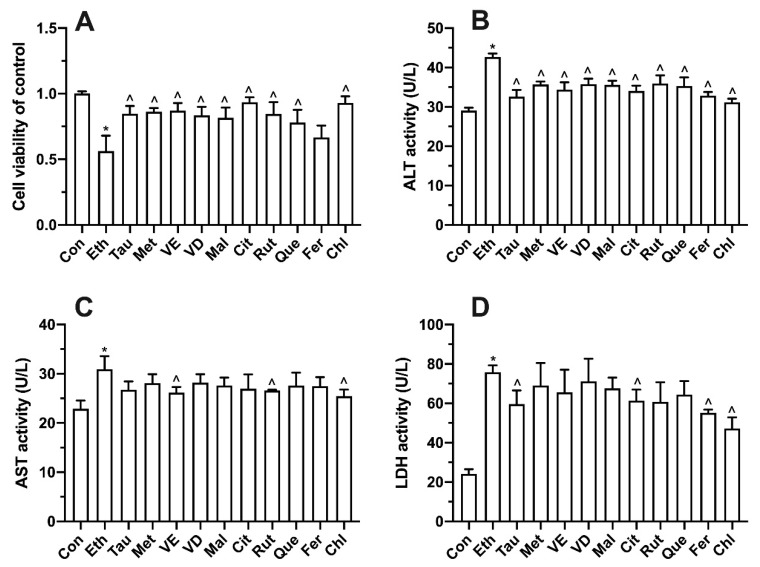
Effect of bioactive compounds on cell viability (**A**), ALT (**B**), AST (**C**), and LDH activity (**D**) in L02 cell lines treated for 100 mM ethanol for 12 h. Values represent means ± SD (n ≥ 6). * *p* < 0.05 compared with control group, ^ *p* < 0.05 compared with 100 mM ethanol. Tau, taurine; Met, methionine; VE, vitamin E; VD, vitamin D; Mal, malic acid; Cit, citric acid; Rut, rutin; Que, quercetin; Fer, ferulic acid; Chl, chlorogenic acid.

As shown in Figure 7A, compared with the control group, the ethanol group showed significantly decreased cell viability. Compared with the ethanol group, all functional component groups had a certain improvement in cell activity but for ferulic acid. Compared with the control group, the activities of ALT, AST and LDH in the ethanol group were significantly increased. All the bioactive compounds significantly decreased the activity of ALT compared with the ethanol group (Figure 7B). Vitamin E, rutin, and chlorogenic acid significantly alleviated the activity of AST compared with the ethanol group (Figure 7C). Taurine, citric acid, ferulic acid, and chlorogenic acid significantly decreased the activity of LDH compared with the ethanol group (Figure 7D).

### 3.8. Effect of Bioactive Components on Intracellular Oxidative Stress and Inflammatory Factors

**Figure 8:**
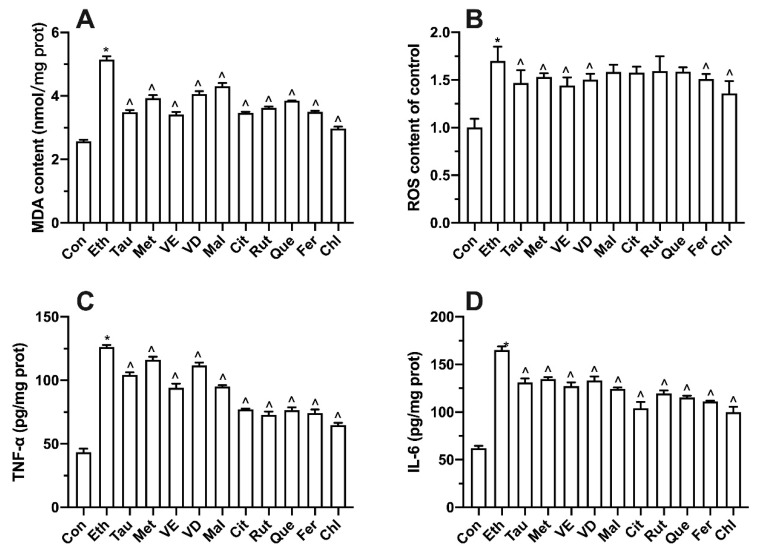
Effect of bioactive compounds on malondialdehyde (**A**), reactive oxygen species (**B**), TNF-α (**C**), and IL-6 (**D**) in L02 cell lines treated for 100 mM ethanol for 12 h. Values represent means ± SD (n ≥ 6). * *p* < 0.05 compared with the control group, ^ *p* < 0.05 compared with the 100 mM ethanol group. Tau, taurine; Met, methionine; VE, vitamin E; VD, vitamin D; Mal, malic acid; Cit, citric acid; Rut, rutin; Que, quercetin; Fer, ferulic acid; Chl, chlorogenic acid.

It can be seen from Figure 8 that compared with the control group, the content of MDA, ROS, TNF-α, and IL-6 was significantly increased in the ethanol group. All bioactive compounds significantly showed alleviation in MDA, TNF-α, and IL-6 level (Figure 8A,C,D). Compared with the ethanol group, taurine, methionine, vitamin E, vitamin D, ferulic acid, and chlorogenic acid significantly decreased the content of ROS (Figure 8B).

### 3.9. Effect of Bioactive Components on Membrane Potential

**Figure 9:**
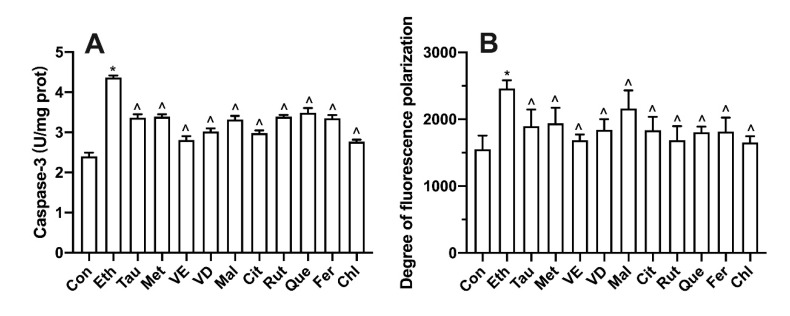
Effect of bioactive compounds on caspase 3 (**A**) and degree of fluorescence polarization (**B**) in L02 cell lines treated for 100 mM ethanol for 12 h. Values represent means ± SD (n ≥ 6). * *p* < 0.05 compared with the control group, ^ *p* < 0.05 compared with 100 mM ethanol. Tau, taurine; Met, methionine; VE, vitamin E; VD, vitamin D; Mal, malic acid; Cit, citric acid; Rut, rutin; Que, quercetin; Fer, ferulic acid; Chl, chlorogenic acid.

The activity of caspase 3 can reflect the index of apoptosis. As shown in Figure 9A, compared with the control group, caspase 3 activity in the ethanol group was significantly increased. However, all functional components significantly decreased the activity of caspase 3. Dibac4 (3) is a kind of probe that can emit fluorescence after binding with proteins in cytoplasm. It can reflect the degree of change in cell membrane potential by measuring the fluorescence intensity. According to the data analysis in Figure 9B, compared with the control group, the fluorescence intensity of cell membrane potential in the ethanol group was significantly increased. However, the fluorescence intensity of cell membrane potential was decreased after treatment with functional components.

## 4. Discussion

According to the experimental data, the degree of cell damage in each group basically increased with the increase in induction time. After 1 h of cell induction, the cell state almost did not change. After 7 h of induction, cell permeability, oxidative stress, lipid peroxidation and inflammation increased, since ethanol and acetaldehyde were completely metabolized and thus the metabolic process caused cell damage (Figure 2) [29]. After 12 h treatment, the degree of cell damage increased, which may be related to the fact that ROS, MDA and inflammatory factors could not be completely removed within 12 h [30]. The trend of ROS production in the acetaldehyde group was similar to that in the ethanol group, which was mainly due to the production of oxygen free radicals by acetaldehyde metabolism (Figure 4) [31]. Proinflammatory cytokines like IL-6 and TNF-α play a significant role in the pathogenesis of alcoholic liver disease [32]. Compared with the ethanol and acetaldehyde groups, the indices of ROS and MDA in the 4-MP groups (unmetabolized ethanol and unmetabolized acetaldehyde) were not that high, indicating that ROS and lipid peroxidation could be produced by ethanol and acetaldehyde in the metabolic process [33]. Based on the experimental results, we finally chose 100 mM ethanol and 12 h of induction as the conditions for the alcoholic liver injury model for subsequent experiments, consistent with the previous studies [34].

Comparing the effects of different functional components, it was found that all functional components could inhibit ethanol-induced apoptosis, which may be related to the structure containing multiple sulfur-containing residues [35]. The effect of vitamin E was superior to vitamin D, which may be related to the fact that the hydroxyl group of vitamin E is a phenolic hydroxyl group, which has higher activity than the common hydroxyl group [36]. The reason that the citric acid group showed better effects on cell activity was that it contains more hydroxyl groups. Among the four polyphenols, chlorogenic acid group showed the best protective effect on cell survival rate, which was consistent with the results of previous studies, since the chlorogenic acid has stronger antioxidant activity than rutin, quercetin and ferulic acid [37].

The protective effect of functional components on cell membrane was mainly reflected in their oxygen free radical scavenging ability and water lipid solubility [38]. The variation trend of ROS content was consistent with that of MDA content, which was mainly due to the oxidation of unsaturated fatty acids by oxygen free radicals [39]. Chlorogenic acid showed the strongest scavenging capacity of oxygen free radicals (Figure 8). On the one hand, the molecular structure of chlorogenic acid contains a large number of hydroxyl groups [40]. On the other hand, ethanol is hydrophilic and lipophilic, which can help chlorogenic acid enter cells and play a role [41]. Compared with amino acids and organic acids, taurine and citric acid showed stronger antioxidant capacity than methionine and malic acid, which was related to more antioxidant groups in structure. Although vitamin E has only one phenolic hydroxyl group [42,43], the activity of phenolic hydroxyl group is much higher than that of the common hydroxyl group, and vitamin E itself can enter the cell membrane, so its ability to prevent cell membrane oxidation was not inferior to that of polyphenols.

With the increase in cell membrane permeability, ALT, AST and LDH released by cells increased correspondingly, which was consistent with the content of MDA [44]. MDA has the function of binding membrane protein, and its increase will limit the flow of cell membrane [45]. As can be seen from the measurement of fluorescence polarization degree of cell membrane fluidity, the group with high MDA content had higher fluorescence polarization degree of cell membrane fluidity, while the viscosity of cell membrane was not conducive to cell flow [46]. The oxygen free radicals produced by the metabolism of ethanol and acetaldehyde are the main cause of cell membrane damage [47]. This causes lipid peroxidation of cell membrane through the formation of intracellular peroxidation, resulting in an increase in membrane permeability and decrease in cell membrane fluidity [48]. Therefore, the antioxidant capacity of functional components can effectively improve the membrane damage induced by ethanol.

In addition to the effects of ethanol metabolism on membrane permeability, membrane fluidity and membrane oxidation and ethanol metabolism can also increase the level of intracellular inflammation [49]. Under the influence of internal and external adverse conditions, cells will secrete TNF-α to participate in systemic inflammatory reaction and can induce apoptosis by producing inflammatory factors such as IL-6 [50]. After 12 h ethanol induction, the trend of intracellular TNF-α level and IL-6 level were promoted, which was similar to the results described by Zhao [51]. Among the functional components, chlorogenic acid showed the best effect in preventing cell inflammation. This may be due to the fact that chlorogenic acid can effectively remove oxygen free radicals and MDA, improve the adverse growth state of the body, thus preventing cell inflammation [52]. The activation of apoptotic protein caspase 3 can promote the apoptosis of severely injured cells and avoid affecting the growth of normal cells [53]. The caspase 3 protein activity in the protection group was similar to the degree of inflammation, indicating that the activation of inflammation would affect cell apoptosis. The experimental results were in line with Yang’s description of the relationship between inflammation and apoptosis [54].

## 5. Conclusions

The effects of ethanol, acetaldehyde, either, and both combined with their metabolism inhibitor on the L02 cell line were studied to analyze the specific components that caused damage to hepatic cells. Results showed that the metabolism of ethanol can promote the content of ROS, MDA, TNF-α, IL-6 and caspase 3, causing oxidative and inflammatory stress, apoptosis, and membrane permeability changes. Amino acids (taurine and methionine), vitamins (vitamin E and vitamin D), organic acids (malic acid and citric acid), flavonoids (rutin and quercetin) and phenolic acids (ferulic acid, and chlorogenic acid) were used to evaluate the protective effects on alcohol-induced cell membrane damage and apoptosis of L02 cells. The conclusion is that chlorogenic acid, taurine, vitamin E and citric acid played an important role in improving cell membrane damage and preventing cell apoptosis, which showed protective effects on hepatocyte damage caused by alcohol.

## Figures and Tables

**Table 1:** The concentration and structure of active compounds used in this study.

Category	Compound	Structure	Concentration (µM)
amino acids	Taurine	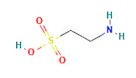	400
	methionine	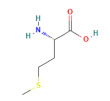	200
vitamins	vitamin E	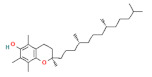	100
	vitamin D	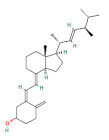	100
organic acids	malic acid	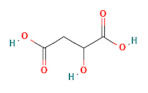	200
	citric acid	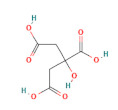	200
flavonoids	rutin	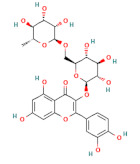	100
	quercetin	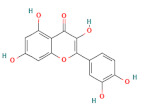	100
phenolic acids	ferulic acid	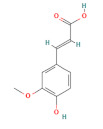	100
	chlorogenic acid	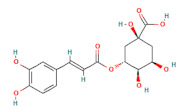	100

## Data Availability

Data is contained within the article.
